# Real-world treatment patterns and outcomes of patients with hormone receptor-positive/HER2-low metastatic breast cancer treated with chemotherapy

**DOI:** 10.1093/oncolo/oyaf106

**Published:** 2025-06-17

**Authors:** Sandhya Mehta, Jackie Kwong, Clara Lam, Bruce Feinberg

**Affiliations:** Daiichi Sankyo, Basking Ridge, NJ, United States; Johnson & Johnson, New Brunswick, NJ, United States; AstraZeneca, Wilmington, DE, NC, United States; Cardinal Health, Real-World Evidence and Insights, Dublin, OH, USA (BF)

**Keywords:** HER2-low, metastatic breast cancer, real-world, chemotherapy

## Abstract

**Introduction:**

Hormonal therapy (HT) based regimen is the preferred first-line (1L) treatment for hormone receptor-positive (HR+) metastatic breast cancer (mBC) with human receptor epidermal growth factor 2 (HER2)-low expression. However, HT resistance frequently emerges with many receiving subsequent chemotherapy (CT). This study aimed to examine CT treatment patterns and outcomes among patients with HR+/HER2-low mBC.

**Patients and methods:**

Patient characteristics and clinical data of adults receiving CT for HR+/HER2-low mBC were collected via physician-abstracted chart review from 10/1/2021 to 1/31/2022. Data were summarized using descriptive statistics with the Kaplan-Meier method to estimate time-to-event outcomes. Statistical comparisons were conducted between patients who received 1L CT vs CT after HT-based regimens (any line).

**Results:**

Two hundred and twenty three HR+/HER2-low patients were included, and CT utilization was described by line within metastatic setting: 1L = 20.2% (*n* = 45), 2L 26.4% (*n* = 59), 3L+ = 53.4% (*n* = 119). A higher rate of visceral metastases (86.7% vs 65.7%, *P* = .01) and lower Eastern Cooperative Oncology Group (ECOG) score (88.9% vs 70.2%, *P* = 0.01) were associated with 1L CT vs CT post-HT-based treatment. The median time-to-treatment discontinuation (TTD) and real-world progression free survival (rwPFS) of CT were similar between the groups (TTD: 6.7 months vs 8.3 months for the 1L CT and CT post-HT-based regimen groups, respectively, *P* = .13; rwPFS: 9.3 months vs 8.8 months, *P* = .26).

**Conclusion:**

In this sample of HR+/HER2-low mBC patients, most patients switched to CT after two lines of therapy with a median rwPFS shorter than 10 months. The findings highlight unmet needs for a more effective targeted therapeutic alternative to CT for HR+/HER2-low mBC patients.

**Implications for practice**

The limited real-world evidence on patients undergoing CT for HR+/HER2-low mBC underscores the significance of this retrospective study of patients receiving CT as 1L therapy or post-HT. Findings underscore the relatively prevalent real-world use of 1L CT and CT post-HT-based treatment in this setting with suboptimal clinical outcomes, emphasizing the imperative for more efficacious therapeutic alternatives than those available during this study period. The advent of HER2-targeted treatment for this indication will likely help to address this. The data prompt a reevaluation of treatment strategies and advocate for the real-world incorporation of novel interventions to improve outcomes.

## Introduction

The human epidermal growth factor receptor 2 (HER2)-low breast cancer, historically classified as HER2-negative^1,2^ accounts for approximately 65% of hormone receptor (HR)-positive (HR+) disease and half of all breast cancer cases.^1,3^ The HER2-low classification is defined by a score of 1+ or 2+ on immunohistochemical (IHC)-based analysis of HER2 protein levels coupled with a lack of HER2 gene (*ERBB2*) amplification as determined via an in situ hybridization (ISH)- based assay (eg, fluorescence in situ hybridization [FISH]).^3,4^ Patients with HER2-low breast cancer generally have a less favorable prognosis as compared to those with HER2-positive disease who are treated with HER2 targeted treatment, with breast cancer specific or overall survival demonstrated to be superior among patients with HER2-positive advanced or metastatic breast cancer than those with HER2-low disease.^5,6^

No HER2-targeted treatment options had demonstrated clinical benefit in the HER2-low metastatic breast cancer (mBC) setting until recently. However, in 2022, the antibody-drug conjugate (ADC) trastuzumab deruxtecan (T-DXd) received approval by the United States (U.S.) Food and Drug Administration (FDA) for the treatment of adults with unresectable breast cancer or mBC previously treated with chemotherapy (CT) in the metastatic setting or who developed disease recurrence during or within 6 months of completing adjuvant CT.^7^ This approval was based on results from the DESTINY-Breast04 Phase III trial in patients with unresectable or metastatic HER2-low disease who received prior CT and hormone therapy (HT) if disease was HR+, which demonstrated improved survival of T-DXd-treated patients vs patients treated with CT only (median progression-free survival: 9.9 months vs 5.1 months, respectively, hazard ratio for disease progression or death, 0.50; *P* < .001).^4^ Since the time that our real-world study was conducted and the National Comprehensive Cancer Network (NCCN) guidelines were updated to include T-DXd as a treatment option for HER2 IHC 1+ or 2+/ISH negative mBC.^8^

Prior to T-DXd approval for this population of HR + HER2low mBC, HR status typically guided treatment decision-making in this setting; hence, HR+/HER2-low and HR-negative/HER2-low disease would generally necessitate distinct therapeutic strategies.^9^ In patients with HR + mBC HT with or without cyclin-dependent kinase 4/6 inhibitors (CDK4/6i) is currently standard of care for first-line (1L) treatment.^9^ Nevertheless, some patients may receive other therapies as 1L therapy in the real-world setting, despite evidence that treatment with therapy prior to CDK4/6i-based treatment is associated with poorer outcomes in comparison to treatment with 1L CDK4/6i-based therapy.^10^ Prescribing patterns vary based on prior adjuvant therapy, timing of metastatic progression, metastatic site and volume, tolerance, and response to prior hormonal therapy (HT), among other factors. Pivotal trials of CDK 4/6 inhibitors (eg, MONARCH (abemaciclib), PALOMA (palbociclib) and MONALEESA (ribociclib)) demonstrated HT alone had a progression-free survival (PFS) of less than one year, while adding a CDK 4/6 inhibitor to HT resulted in a PFS of nearly two years.^11^ Since most mBC patients survive initial hormone-based treatment, nearly all will receive at least one line of CT.^12^

Endocrine resistance occurs due to several molecular mechanisms and is defined per the international guidelines as primary [as a relapse within two years while on adjuvant HT-based treatment or disease progression within the first six months of 1L HT-based treatment for mBC] or secondary resistance [as a relapse while on adjuvant HT-based treatment but after first two years or within the first year of completing adjuvant endocrine therapy or progression ≥ six-months after initiating 1L HT-based treatment for mBC.^13^ Among patients receiving an HT-based regimen, endocrine resistance frequently emerges as early as two years with an incidence of 20% to 25%, and many patients subsequently receive CT.^13-15^

Given the fact that a high proportion (50%-60%) of HR + mBC is characterized by HER2-low expression,^2^ and that prognosis is generally poor among the HER2-low population relative to HER2-positive patients,^5,6^ understanding the patient characteristics, current treatment patterns, and clinical outcomes of patients with HR+/HER2-low mBC treated in the real-world setting is paramount in the development of innovative, effective therapies. However, data specific to the treatment journey and clinical outcomes of patients with HR+/HER2-low mBC treated in the real-world setting are limited. The primary objective of this analysis was to describe the characteristics, treatment patterns, and outcomes of U.S. patients treated with CT for HR+/HER2-low mBC in a real-world setting. We specifically assessed differences in characteristics and outcomes among patients who received CT as their 1L treatment vs those who received CT following an HT-based regimen.

## Methods

### Data source and study design

A retrospective, observational, U.S. multi-site, physician-abstracted medical chart review cohort study of adults with HR+/HER2-low mBC (primary group of interest for this analysis), HR-negative (HR-)/HER2-low mBC, and HER2 immunohistochemical (IHC) 0 mBC was conducted. Subgroup comparison of patients with HR+/HER2-low disease who received CT as 1L treatment or following an HT-based regimen was conducted and is the focus of this analysis. The research was conducted via the Cardinal Health Oncology Physician Extended Network (OPEN) which consists of approximately 850 specialty certified practicing physicians from the real-world research community who have contributed patient-level data via chart abstraction since its inception in 2019. Physicians used structured data elements and unstructured clinical notes within the electronic medical records (EMRs) as well as uploaded PDFs and external hospital records to collect objective data. The data were supplemented with physician observations and perceptions. The physicians were compensated according to fair market value for data abstraction time based on an electronic case report form (eCRF) beta test by four of their peers.

The participating physicians provided information about their practice characteristics and abstracted structured and unstructured data from medical records related to patient demographics, clinical characteristics, treatment patterns, and clinical outcomes in an eCRF. Key data elements collected in the eCRF are presented in **Supplementary Table S1**. Each physician could submit a maximum of 10 eCRFs and were instructed to select patients starting with the first (earliest) eligible patient and select subsequent patients consecutively in chronologic order. Quotas were established for the maximum number of patients that could be submitted by any physician in groups based on HR status, HER2 status, or treatment received in an effort to obtain sufficient patient numbers for subgroup analyses.

Data abstraction originally took place between 10/1/2021 and 12/10/2021, with secondary data collection aimed to collect data for additional patients occurring between 1/4/2022 and 1/31/2022. The Cardinal Health research operations team conducted content and data quality checks throughout the research process as described in **Supplementary Table S2**. The study received approval and exemption for obtaining patient informed consent by a central Institutional Review Board (IRB) and followed all standard research guidelines.

### Study population and cohort selection

Eligible patients with mBC were age 18 years and older at diagnosis with mBC and received CT for mBC. All patients included in the study had documented HER2 and HR status. Patients with HR +  disease must have had at least three lines of therapy, with the 1L initiated between 02/19/2016, and 12/31/2018. Additional eligibility details are described in **Supplementary Table S3**. Patients with HER2 IHC scores of 1+, or IHC 2+ and with a negative test result for *ERBB2* amplification via FISH/ISH testing were classified as having HER2-low disease. Patients who participated in any clinical trial for mBC were excluded. For the purpose of the analyses presented here, the HR+/HER2-low cohort was further broken down by treatment received, with key subgroups including those who received CT as 1L therapy for mBC and those who received CT following an HT-based regimen. Key patient subgroups are presented in a flow chart (**Supplementary Figure S1**).

### Statistical analysis

Baseline characteristics and treatment patterns were summarized using descriptive statistics. The two comparison groups were 1L CT (*n* = 45) vs CT following an HT-based regimen (*n* = 178). In the context of these analyses, CT index therapy was defined as 1L therapy for the “1L CT” group and as the first CT received following an HT-based regimen for the “CT after an HT-based regimen” group. Real-world progression-free survival (rwPFS) and real-world time-to-discontinuation (TTD) were described using the Kaplan-Meier method, accounting for right-censoring. rwPFS was defined as time from initiation of therapy to first reported disease progression or death. Patients who discontinued treatment for a reason other than disease progression or death were censored at the discontinuation date and patients still on treatment at the time of data collection were censored at the date of last encounter. TTD was defined as the time from the initiation to the date of discontinuation of a particular regimen or date of death, whichever occurred first. Patients who did not discontinue therapy were censored at the last encounter. Baseline characteristics and outcomes were compared between subgroups using the chi-square test, Fisher’s exact test *t*-test, or log-rank test, depending on the variable or endpoint type. Cox proportional hazards (PH) regression was used to investigate the association of baseline demographic and clinical variables with rwPFS and to investigate the difference in rwPFS associated with 1L CT vs CT after an HT-based regimen groups after adjusting for patient demographic and clinical variables. Statistical analyses were performed using the Statistical Analysis Software (SAS) (SAS software version 9.4, SAS Institute Inc.). A 2-sided *P* < .05 was considered statistically significant.

## Results

### Demographics and clinical characteristics

A total of 223 patients with HR+/HER2-low mBC (the primary population of interest for the analysis presented here) were included in a wider real-world study of 444 patients with HER2 IHC0 or HER2-low mBC, with data submitted by 33 oncologists (primarily community practitioners). The characteristics of participating physicians are presented in **Supplementary Table S4** and baseline demographic and clinical characteristics of all patients included in the wider-real-world study are detailed in **Supplementary Table S5**. No statistically significant differences were observed between the HER2-low and HER2 IHC0 populations in the wider real-world study related to treatment patterns (**Supplementary Table S6**) and characteristics.

The baseline characteristics of patients with HR+/HER2-low disease included in this analysis are presented in Table 1 and **Supplementary Table S7**. All patients in this analysis were female and had a median age at mBC diagnosis of approximately 60 years old. The study cohort was 62% White Americans, 27% Black American, and half of the patients had Medicare insurance. Overall, patient demographics were similar between subgroups based on receipt of CT as 1L therapy vs following an HT-based regimen in terms of age at mBC diagnosis (*P* = .92), race (*P* = .07), ethnicity (*P* = .23), and insurance status (*P* = .68). Among the 223 patients studied, 142 were diagnosed with de novo metastatic disease and underwent HER2 IHC testing at the time of metastatic diagnosis. The remaining 81 patients presented with stage 1-3 disease and were tested at the time of their initial diagnosis.

**Table 1.** Patient characteristics and duration of follow-up among patients with HR+/HER2-low^1^ disease.

	HR+/HER2-low^1^	1L CT	CT After an HT-based regimen	
	*n* = 223	*n* = 45	*n* = 178	P
Sex at birth, female, *n* (%)	223 (100.0)	45 (100.0)	178 (100.0)	–
Age at mBC diagnosis, mean (SD) years	60.4 (11.1)	60.1 (11.9)	60.5 (11.0)	.92^a^
Race, *n* (%)				.07^b^
White	138 (61.9)	28 (62.2)	110 (61.8)	
Black/African American	61 (27.4)	10 (22.2)	51 (28.7)	
Asian	15 (6.7)	2 (4.4)	13 (7.3)	
Native Hawaiian or Other Pacific Islander	1 (0.4)	0 (0.0)	1 (0.6)	
American Indian or Alaska Native	1 (0.4)	1 (2.2)	0 (0.0)	
Unknown^2^	7 (3.1)	4 (8.9)	3 (1.7)	
Ethnicity (*n*, %)				.23^b^
Hispanic/Latino/Latina	29 (13.0)	9 (20.0)	20 (11.2)	
Non-Hispanic/Non-Latino/Non-Latina	192 (86.1)	36 (80.0)	156 (87.6)	
Unknown	2 (0.9)	0 (0.0)	2 (1.1)	
Most recent primary insurance (*n*, %)				.68^b^
Medicare	111 (49.8)	21 (46.7)	90 (50.6)	
Commercial	93 (41.7)	22 (48.9)	71 (39.9)	
Medicaid	17 (7.6)	2 (4.4)	15 (8.4)	
Self-pay	1 (0.4)	0 (0.0)	1 (0.6)	
Other (“Medicare Advantage” entered as free text)	1 (0.4)	0 (0.0)	1 (0.6)	
Patient U.S. residence region (*n*, %)^3^				<.01^c^
Northeast	57 (25.6)	25 (55.6)	32 (18.0)	
Midwest	38 (17.0)	2 (4.4)	36 (20.2)	
South	75 (33.6)	4 (8.9)	71 (39.9)	
West	53 (23.8)	14 (31.1)	39 (21.9)	
Anatomic site of metastasis at diagnosis of mBC (*n*, %)^4^^,^^5^				
Visceral^6^	164 (73.5)	39 (86.7)	117 (65.7)	.01^c^
Adrenal gland	22 (9.9)	5 (11.1)	17 (9.6)	.78^b^
Brain	13 (5.8)	7 (15.6)	6 (3.4)	.01^b^
Gastrointestinal system	5 (2.2)	1 (2.2)	4 (2.2)	1.0^b^
Ovary	5 (2.2)	1 (2.2)	4 (2.2)	1.0^b^
Liver	73 (32.7)	31 (68.9)	42 (23.6)	<.01^c^
Lung	113 (50.7)	24 (53.3)	89 (50.0)	.69^c^
Pleura, pericardial, and/or peritoneal cavity	27 (12.1)	10 (22.2)	17 (9.6)	.02^c^
Non-visceral	195 (87.4)	36 (80.0)	149 (83.7)	.55^c^
Bone	159 (71.3)	26 (57.8)	133 (74.7)	.02^c^
Skin/soft tissue	18 (8.1)	4 (8.9)	14 (7.9)	.76^b^
Local lymph node(s)	34 (15.2)	8 (17.8)	26 (14.6)	.60^c^
Regional/Distal lymph node(s)	53 (23.8)	18 (40.0)	35 (19.7)	<.01^c^
Bone marrow (entered as free text under “other”	1 (0.4)	1 (2.2)	0 (0.0)	.20^b^
Number of distinct anatomic sites of metastasis at diagnosis of mBC (*n*, %)				
0 to 1	65 (29.1)	7 (15.6)	58 (32.6)	<.01^c^
2 to 3	125 (56.1)	24 (53.3)	101 (56.7)	
4+	33 (14.8)	14 (31.1)	19 (10.7)	
ECOG-PS at CT index*, *n* (%)				
0/1	165 (74.0)	40 (88.9)	125 (70.2)	.01^c^
2+	58 (26.0)	5 (11.1)	53 (29.8)	
Duration of follow-up since initiation of CT index* (months)				
Mean (SD)	16.7 (15.3)	40.3 (10.0)	10.7 (9.5)	<.01^a^
Range	< 1-68.5	11.3-68.5	< 1-45.7	
Median (IQR)	11.3 (4.4-26.1)	39.8 (36.3-42.3)	7.6 (3.5-15.5)	

*CT index was defined as 1L therapy for the 1L CT group and as the first CT received following an HT-based regimen for the CT after an HT-based regimen group. Patients who received CT post CDK4/6i were excluded.

^1^HER2-low defined as IHC 1 + or IHC 2 + and FISH/ISH negative.

^2^Race was entered as Hispanic via open text. However, Hispanic is considered as ethnicity rather than race, and therefore race is unknown.

^3^Northeast includes Connecticut, Delaware, Massachusetts, Maine, Maryland, New Hampshire, New Jersey, New York, Pennsylvania, Rhode Island, Vermont; Midwest includes Iowa, Illinois, Indiana, Kansas, Michigan, Minnesota, Missouri, North Dakota, Nebraska, Ohio, South Dakota, Wisconsin; South includes Alabama, Arkansas, District of Columbia, Florida, Georgia, Kentucky, Louisiana, Mississippi, North Carolina, Oklahoma, South Carolina, Tennessee, Texas, Virginia, West Virginia; West includes Alaska, Arizona, California, Colorado, Hawaii, Idaho, Montana, New Mexico, Nevada, Oregon, Utah, Washington, Wyoming.

^4^Not mutually exclusive.

^5^Additional response options that were presented to physicians but were not selected for any patients are excluded here.

^6^Defined as metastases in the adrenal gland; GI system; GU system; ovary, gynecological system; brain; liver; lung; pleura, pericardial, and/or peritoneal cavity.

^a^ *T* test-pooled test was used for statistical comparison.

^b^Fisher’s Exact test was used for statistical comparison.

^c^Chi-Square test was used for statistical comparison.

Abbreviations: 1L, first line; CDK4/6i, cyclin-dependent kinase 4/6 inhibitor; CT, chemotherapy; ECOG-PS, Eastern Cooperative Oncology Group performance status; FISH, fluorescence in situ hybridization; GI, gastrointestinal tract; GU, genitourinary; HER2, human epidermal growth factor receptor 2; HR+, hormone-receptor positive; IHC, immunohistochemical; IQR, interquartile range; ISH, in situ hybridization; mBC, metastatic breast cancer; SD, standard deviation; U.S., United States.

In comparison to patients who received CT following an HT-based regimen, a greater proportion of patients who received 1L CT were from the Northeast (55.6% vs 18.0%, *P* < .01). The median follow-up duration since initiating CT index was 39.8 (range: 11.3-68.5) months for the 1L CT group and 7.6 (range: <1.0-45.7) months for patients who received CT after an HT-based regimen (*P* < .01).

Patients who received 1L CT had a higher rate of visceral metastases than that of patients who received CT after an HT-based regimen (86.7% vs 65.7%, respectively, *P* = 0.01). In comparison to patients who received CT after an HT-based regimen, at the time of mBC diagnosis, a higher proportion of patients who received 1L CT had metastases to the brain (15.6% vs 3.4%, *P* = .01), liver (68.9% vs 23.6%, *P* < .01), regional/distal lymph node(s) (40.0% vs 19.7%, *P* < .01) and pleura, pericardial, and/or peritoneal cavity (22.2% vs 9.6%, *P* = .02) and a lower proportion of the 1L CT group had bone metastases (57.8% vs 74.7%, *P* = .02). Patients who received 1L CT had a higher number of distinct anatomic sites of metastasis than those who received CT after an HT-based regimen (*P* < .01) (four or more distinct sites: 31.1% vs 10.7%).

A smaller proportion of those who received 1L CT had an Eastern Cooperative Oncology Group performance status (ECOG-PS) score of 2 or more prior to first use of CT than patients who received CT after an HT-based regimen. (11.1% vs 29.8%, *P* = .01). More than half of the patients had hypertension at diagnosis with mBC, but there was no difference in rate of hypertension between the 2 groups (*P* = .26). In addition, no differences in the rates of other comorbidities were observed between the groups. Smoking status at diagnosis with mBC was also similar between subgroups based on 1L CT utilization or CT following an HT-based regimen, and less than half of patients in these groups were current smokers or had a history of smoking.

### Treatment patterns

**Figure 1.**
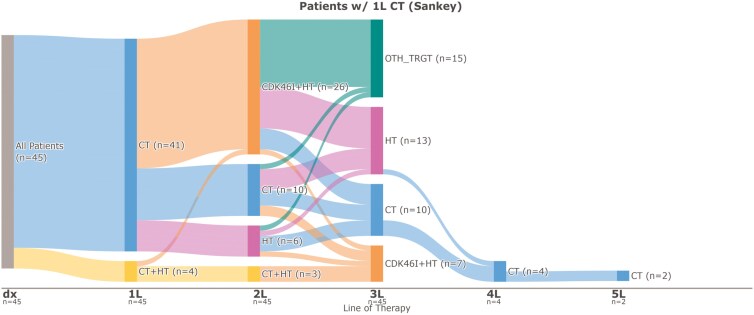
Sankey plot of patients with HR+/HER2-low mBC Who Received 1L CT, *n* = 45. Abbreviations: 1L, first line; 2L, second line; 3L, third line; 4L, fourth line; 5L, fifth line; CDK46_INH, cyclin-dependent kinase 4/6 inhibitor; CT, chemotherapy; dx, diagnosis; HER2, human epidermal growth factor receptor 2; HR+= hormone-receptor positive; HT, hormonal therapy; mBC, metastatic breast cancer; OTH_TRGT, other targeted therapy.

Figure 1 illustrates the patient treatment journey among patients with HR+/HER2-low mBC who received CT as 1L. In this group, 41 patients (91.1%) received CT alone as 1L and 4 patients (8.9%) received CT and an HT-based regimen. Of the 41 patients who received CT only as 1L, the majority (61.0%) went on to receive cyclin-dependent kinase 4/6 (CDK4/6)-targeted therapy and an HT-based regimen as second-line (2L), while 24.4% received CT and 14.6% received an HT-based regimen in 2L. Three of 4 patients who received CT and an HT-based regimen as 1L received CT and an HT-based regimen in 2L. Third-line (3L) treatments received included HT-based therapy, CT, CDK4/6-targeted therapy with HT, and other targeted therapies with or without HT (alpelisib, everolimus, olaparib, sacituzumab govitecan-hzly). Several patients returned to CT treatment following discontinuation of 2L HT-based regimen. Three patients (6.7%) continued to receive additional CT in both 2L and 3L and 1 across 5 lines of therapy.

**Figure 2.**
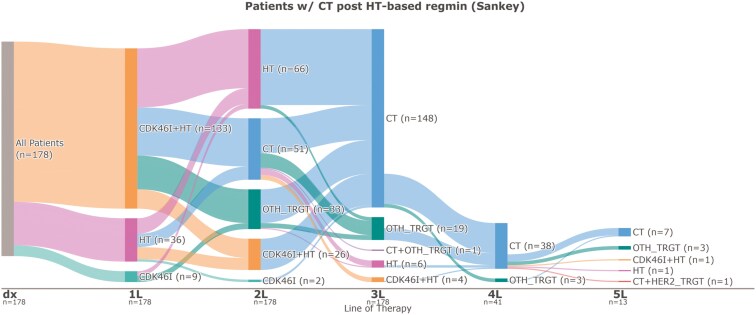
Sankey plot of patients with HR+/HER2-low mBC who received CT after an HT-based regimen, *n* = 178. Abbreviations: 1L, first line; 2L, second line; 3L, third line; 4L, fourth line; 5L, fifth line; CDK46_INH, cyclin-dependent kinase 4/6 inhibitor; CT, chemotherapy; dx, diagnosis; HER2, human epidermal growth factor receptor 2; HR, hormone receptor; HT, hormonal therapy; LOT, lines of treatment; mBC, metastatic breast cancer; OTH_TRGT, other targeted therapy.

The patient treatment journey of 178 patients with HR+/HER2-low mBC who received CT after an HT-based regimen is depicted in **Figure 2**. The majority of patients, 33 (74.7%) in this subgroup received CDK4/6-targeted therapy and HT as 1L therapy, with the remaining patients receiving HT-based treatment, 36 (20.2%) or CDK4/6-targeted therapy alone, 9 (5.1%). Approximately, 51 (28.7%) received CT index as 2L therapy, 119 (66.9%) as 3L, and 8 (4.5%) as fourth line (4L). Among those who received 2L CT (*n* = 51), 24 (47.1%) received CT as either 3L or 4L treatment.

**Figure 3:**
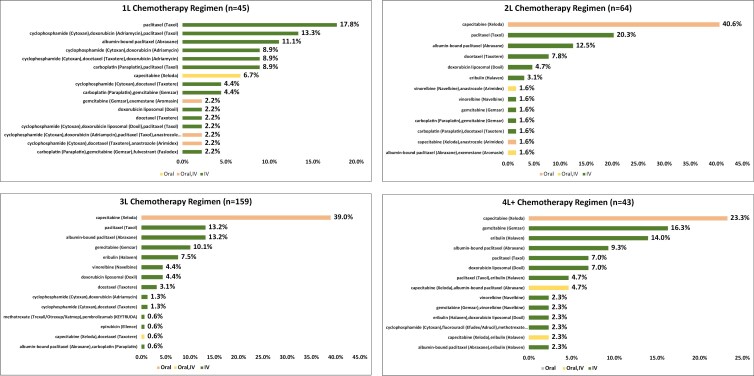
Chemotherapy regimens by line of therapy. Abbreviations: 1L, first line; 2L, second line; 3L, third line; 4L+= fourth line or higher; IV, intravenous.

The CT regimens utilized for each line of therapy are presented in **Figure 3**. Paclitaxel, administered intravenously, was the most frequently used CT regimen in 1L (among 17.8% of patients who received 1L CT), while capecitabine (orally administered) was the most frequently utilized 2L (40.6%), 3L (39.0%), and 4L + (23.3%) CT.

### Clinical outcomes

The clinical outcomes associated with 1L CT vs CT after an HT-based regimen are presented in **Table 2**. Within the follow-up time since initiating CT index (39.8 months and 7.6 months for the 1L CT and CT after an HT-based regimen groups, respectively), the Kaplan Meier estimated rwPFS median was 9.3 (95% confidence interval [CI], 6.7-11.5) months for the 1L CT group and 8.8 (95% CI: 7.7-9.7) months for the CT after an HT-based regimen group, with no statistical difference observed between the subgroups (*P* = .26). The median TTD was 6.7 (95% CI: 5.8-7.1) months and 8.3 (95% CI, 7.6-9.2) months for the 1L CT and CT after an HT-based regimen groups, respectively, again with no difference in median TTD observed between the groups (*P* = .13).

**Table 2.** Clinical outcomes of patients with HR+/HER2-low^1^ disease.

	HR+/HER2-low^1^	Index CT^2^		*P* ^4^
		1L CT	CT After an HT-based regimen	
	*n* = 223	*n* = 45	*n* = 178	
Patients alive at data collection (*n*, %)	157 (70.4)	37 (82.2)	120 (67.4)	.05^a^
rwPFS, months^3^				
No. of censored observations	86 (38.6)	4 (8.9)	82 (46.1)	
No. of events	137 (61.4)	41 (91.1)	96 (53.9)	
Median (95%CI)	8.8 (7.7-9.7)	9.3 (6.7-11.5)	8.8 (7.7-9.7)	.26^b^
TTD, months^4^				
Censored, *n* (%)	78 (35.0)	0 (0.0)	78 (43.8)	
Events, *n* (%)	145 (65.0)	45 (100.0)	100 (56.2)	
Median, (95% CI)	7.7 (7.0-8.6)	6.7 (5.8-7.1)	8.3 (7.6-9.2)	.13^b^
**Discontinuation of LOT** ^5^				
Patients discontinued therapy (*n*, %)	145 (65.0)	45 (100.0)	100 (56.2)	
Reason for discontinuation of line of therapy (*n*, % of patients who discontinued)				
Disease progression (defined clinically)	26 (17.9)	6 (13.3)	20 (20.0)	<.01^c^
Disease progression (confirmed with scan)	84 (57.9)	26 (57.8)	58 (58.0)	
Scheduled duration of therapy complete	10 (6.9)	9 (20.0)	1 (1.0)	
Toxicity/intolerability	4 (2.8)	1 (2.2)	3 (3.0)	
Patient choice	10 (6.9)	3 (6.7)	7 (7.0)	
Death	11 (7.6)	0 (0)	11 (11.0)	

^1^HER2-low defined as IHC 1 + or IHC 2 + and FISH/ISH negative. Time-to event endpoints are reported for 1L therapy.

^2^Defined as any CT received as monotherapy or in combination with another therapy as 1L therapy for the “1L CT” group and as the first CT received following an HT-based regimen for the “CT after an HT-based regimen” group. Time-to event endpoints and ORR are reported for 1L therapy for the “1L CT” group and as the first CT received following an HT-based regimen for the “CT after an HT-based regimen” group.

^3^Kaplan-Meier estimate of time from initiation of therapy to first reported disease progression or death. Patients who discontinued treatment for a reason other than disease progression or death were censored at discontinuation date and patients still on treatment at the time of data collection were censored at date of last encounter.

^4^Kaplan-Meier estimate from initiation to discontinuation of line of therapy or date of death, whichever occurred first. Patients who did not discontinue therapy were censored at last encounter.

^5^Reported for 1L therapy for HR+/HER-2-low cohort, for 1L therapy for the “1L CT” were censored at discontinuation date and patients still on treatment at the time of data collection were censored at date of last encounter.

^a^Chi-square.

^b^Log-rank.

^c^Fisher’s exact.

Abbreviations: 1L, first line; CI, confidence interval; CT, chemotherapy; FISH, fluorescence in situ hybridization; HER2, human epidermal growth factor receptor 2; HR+= hormone-receptor positive; HT, hormonal therapy; IHC, immunohistochemical; ISH, in situ hybridization; LOT, lines of treatment; No.= number; ORR, overall response rate; rwPFS, real-world progression-free survival; TTD, time to treatment discontinuation.

The distribution among responses for reason for CT index discontinuation was distinct between the two subgroups (*P* < .01) (Table 2). Among patients that discontinued CT index, the most common reason for discontinuation was disease progression confirmed with scan (57.8% vs 58.0% among the 1L CT vs CT after an HT-based regimen groups, respectively). In comparison to patients who received CT after an HT-based regimen, a lower proportion of patients who received 1L CT discontinued due to disease progression defined clinically (13.3% vs 20.0%) or death (0.0% vs 11.0%), and a higher percentage discontinued due to the scheduled duration of therapy completion (20.0% vs 1.0%). Kaplan-Meier curves for rwPFS are presented in **Supplementary Figure S2** and for TTD in **Supplementary Figure S3**.

To identify patient characteristics associated with rwPFS, Cox PH regression analyses were performed, as depicted in Table 3. In multivariable analyses, the model adjusted for variables that were statistically significant in the model with the exception of whether CT was received as 1L or after an HT-based regimen, which was retained. An ECOG-PS of 2 or greater at initiation of index CT was the only variable associated with a significantly higher risk of progression or death across all univariable and multivariable Cox PH model analyses conducted (rwPFS hazard ratio for multivariable model: 1.91; 95% CI, 1.28 to 2.85, *P* < .01). Importantly, 1L CT treatment was not associated with higher or lower risk of progression or death than CT treatment after an HT-based regimen.

**Table 3:** Cox proportional hazard regression for characteristics associated with rwPFS among patients with HR+/HER2-low mBC, *n* = 221.

	Unadjusted		Multivariable model^1^	
	HR (95% CI)	*P*	HR (95% CI)	*P*
**Demographics**				
Age at mBC diagnosis (continuous)	1.00 (0.98-1.02)	.77		
Race				
Black/African American race, *n* = 61 (reference: White, *n* = 136)^2^	1.09 (0.75-1.60)	.65		
Other/Unknown race, *n* = 24 (reference: White, *n* = 136)^2^	0.81 (0.46-1.44)	.48		
Hispanic ethnicity, *n* = 29 (reference: non-Hispanic, *n* = 192)^3^	1.02 (0.60-1.70)	.95		
Place of U.S residence				
Midwest *n* = 38 (reference: South, *n* = 75)	0.96 (0.56-1.65)	.88		
Northeast *n* = 57 (reference: South, *n* = 75)	0.87 (0.56-1.35)	.54		
West *n* = 51 (reference: South, *n* = 75)	1.14 (0.71-1.83)	.60		
Most recent primary insurance provider				
Medicaid, *n* = 17 (reference: Medicare, *n* = 111)	0.88 (0.44-1.78)	.73		
Commercial or Self-pay, *n* = 93 (reference: Medicare, *n* = 111)^4^	0.94 (0.66-1.34)	.71		
**Clinical characteristics/medical history**				
Stage IV at initial diagnosis, *n* = 142 (reference: Stage I-III, *n* = 79)^5^	0.86 (0.61-1.22)	.40		
Current or past smoker, *n* = 101 (reference: never smoked, *n* = 120)	0.94 (0.67-1.33)	.73		
ECOG-PS 2 + at initiation of index CT, *n* = 57 (reference: ECOG-PS of 0 or 1, *n* = 164)	1.95 (1.31-2.90)	<.01	1.91 (1.28-2.85)	<.01
Local lymph node involvement, yes, *n* = 34 (reference: no, *n* = 187)	1.20 (0.77-1.88)	.41		
Regional lymph node involvement, yes, *n* = 53 (reference: no, *n* = 168)	1.11 (0.74-1.67)	.61		
2 + distinct anatomic sites of metastasis, *n* = 156 (reference: 0 to 1, *n* = 65)	1.05 (0.70-1.56)	.83		
Visceral involvement, yes, *n* = 154 (reference: no, *n* = 67)^6^	1.01 (0.67-1.52)	.97		
Brain metastases, yes, *n* = 13 (reference: no, *n* = 208)	0.72 (0.39-1.35)	.31		
Bone metastases, yes, *n* = 157 (reference: no, *n* = 64)	1.28 (0.89-1.85)	.18		
Pleura, pericardial, and/or peritoneal cavity metastases, yes, *n* = 26 (reference: no, *n* = 195)	1.16 (0.73-1.85)	.54		
Liver metastases, yes, *n* = 72 (reference: no, *n* = 149)	0.93 (0.65-1.32)	.67		
History of hypertension, yes, *n* = 127 (reference: no, *n* = 94)	1.17 (0.83-1.66)	.37		
History of cardiovascular disease/congestive heart failure/myocardial infarction, yes, *n* = 47 (reference: no, *n* = 174)	1.57 (1.05-2.34)	.03		
History of depression, yes, *n* = 41 (reference: no, *n* = 180)	1.01 (0.66-1.55)	.97		
History of diabetes, yes, *n* = 65 (reference: no, *n* = 156)	0.83 (0.57-1.20)	.31		
History of renal disease, yes, *n* = 26 (reference: no, *n* = 195)	1.13 (0.70-1.84)	.62		
**Treatment**				
1L CT, *n* = 45 (reference: first CT after an HT-based regimen, *n* = 176)	0.81 (0.55-1.19)	.29	0.88 (0.59-1.30)	.51

^1^The proposed model was built using a combination of forward, backward, stepwise, and manual addition/deletion of variables to only keep variables that were statistically significant in the model with the exception of whether CT was received as 1L or after an HT-based regimen, which was retained in the final model.

^2^Seven patients had unknown race and all these patients had ethnicity listed as Hispanic. These patients were grouped with Non-White.

^3^Two patients had unknown ethnicity. Both patients had Black or African American as their race. These patients were grouped with non-Hispanic patients.

^4^One patient had Self-pay as payer.

^5^Two patients had unknown stage; these patients were excluded from the analysis. Both were from CT after an HT-based regimen group.

^6^Visceral metastases are defined as metastases in the adrenal gland; GI system; GU system; ovary, gynecological system; brain; liver; lung; pleura, pericardial, and/or peritoneal cavity.

**Abbreviations:** 1L, first line; CI, confidence interval; CT, chemotherapy; ECOG-PS, Eastern Cooperative Oncology Group-performance status; GI, gastrointestinal tract; GU, genitourinary; HR, hazard ratio; HR+= hormone-receptor positive; HER2, human epidermal growth factor receptor 2; HR, hormone receptor; HT, hormonal therapy; mBC, metastatic breast cancer; rwPFS, progression-free survival.

## Discussion

This analysis from a retrospective, multi-site, United S.tates, primarily community oncology-based physician-abstracted medical chart review study describes the demographics, clinical characteristics, treatment patterns, and clinical outcomes of patients who received 1L CT or CT after an HT-based regimen for HR+ /HER2-low mBC as a part of routine clinical care. Our real-world investigation revealed several key findings.

First, in the real-world patient population included in this analysis, CT utilization increased in later lines of treatment, with the majority of patients receiving CT after 2 lines of prior HT-based regimen, which is accordance to the NCCN guidelines.^8^ Switching from an HT-based regimen to CT may be due to the development of endocrine resistance,^13,16^ though other reasons for switching cannot be ruled out. Primary endocrine resistance has typically been defined in the literature as a relapse within 2 years of adjuvant endocrine treatment or disease progression during the first 6 months of 1L endocrine therapy for advanced or mBC.^13^ Future research is needed to understand how physicians define endocrine resistance on a case-by-case basis in the real-world practice setting and rationale for switching to CT.

Approximately one-fifth of our real-world cohort of patients with HR+/HER2-low mBC described in this analysis received 1L CT, the majority of whom had visceral sites of metastasis and higher disease burden as measured by number of metastatic sites. This proportion is smaller than that of previously published real-world data in the HR+/HER2-low mBC setting from 10 or more years ago.^17,18^ Hence, 1L CT usage in the US real-world setting appears to be decreasing and likely associated primarily with visceral crisis and high disease burden. This may indicate that community physicians base their determination of 1L treatment strategy, in part, on the presence of visceral metastases and perhaps other indicators of poor prognosis. Guidelines indicate that incidence of visceral crises (visceral metastases and severe organ dysfunction) requires CT over an HT-based regimen.^19^ CT is recommended in these patients as an urgent need to achieve the greatest possible tumor response in the shortest time.^20^ However, the treatment patterns among those with aggressive disease are likely to change with recently published results from “RIGHT choice” trial showcasing clinical benefit of ribociclib + HT vs CT.^21^ Conversely, patients who received 1L CT generally had more favorable ECOG-PS scores prior to CT initiation and lower incidence of bone metastases at mBC diagnosis than those who received CT after HT. This suggests a potential association between the choice of 1L treatment and existing bone metastasis, shedding light on the nuanced impact of different therapeutic strategies and metastatic profiles. ECOG-PS scale measures the ability to tolerate cytotoxic CT and impact on survival and a score>2 indicates poor tolerability to cytotoxic CT.^20^ The superior performance status observed in the 1L CT group as measured by the ECOG-PS scale, emphasizes the relevance of this metric in gauging tolerance to cytotoxic CT and its impact on overall survival.^20^

In this study, the median TTD and rwPFS associated with CT were under 10 months and similar between patients who received 1L CT and those who received CT following an HT-based regimen. However, differences in sample size and duration of follow-up time between these subgroups should be considered when interpreting this data, with shorter median follow-up observed for the subgroup of patients who received CT after HT-based regimens. Published data on clinical outcomes associated with CT following endocrine resistance in HR+/HER2-low or HER2-negative mBC is extremely limited, with one published study reporting median time-to-treatment failure of approximately 6 months or less for chemotherapy following endocrine resistance in HR+/HER2-negative mBC.^22^ However, clinical trials have demonstrated a median time to progression of CT in later lines (2L or greater) ranging from 4.1 to 4.9 months.^2,23,24^ Despite differences in populations between the clinical trial and real-world setting, the real-world evidence on CT outcomes generally aligns with that of clinical trials. All together, these findings highlight the need for more effective therapy post-endocrine-based regimens in this setting.

New treatment options in this setting such as the HER2-targeting therapy, T-DXd,^25^ may be more effective for certain patients with disease that progressed following HT-based regimens. Additionally, future research may identify interactions between hormone receptors and HER2 receptors that provide insight into mechanisms of treatment resistance in HER2-low mBC. Further research is warranted to evaluate the safety and effectiveness of new treatment options such as T-DXd in the real-world setting.

### Limitations

While providing valuable insights, our study is not without limitations. This study employed purposive sampling, with selection based on pre-specified criteria; hence, findings may not be representative of treatment patterns of all physicians managing patients with this indication or of all patients with this indication. The modest sample size underscores the necessity for larger-scale investigations to enhance the generalizability of our findings. Furthermore, it is important to note that certain patient characteristics were not encompassed in the data collection, thereby remaining unaccounted for in the analyses. Further, loss to follow-up may have occurred in cases of transfer of care outside of the physician’s practice, with potentially missing information following the date of last visit. Follow-up time since CT index was particularly limited for the CT after an HT-based regimen group (7.6 months) and additional follow-up may help to improve estimates of clinical outcomes. Notably, over 70% of all patients were still alive at the time of data collection.

Importantly, the median follow-up time was significantly longer among patients who received 1L CT than those who received CT post-HT-based treatment, and a higher rate of censoring was conducted for the latter, which should be considered when interpreting findings from Kaplan-Meier-based estimates for the two groups. Additionally, the retrospective nature of the study poses inherent limitations, requiring prospective studies to validate the observed treatment patterns and outcomes. Finally, results may be impacted by inaccuracies in certain data elements collected, including reported levels of HER2 expression, as measured by IHC and FISH/ISH.^26^ Given the perceived lack of clinical utility of accurately distinguishing IHC scores of 0 and 1 + in the past,^27^ the reliability, precision, and accuracy of historical IHC0 and IHC1 + scores may potentially be questionable. The complexity of mBC and the evolving landscape of treatment options further warrant continuous scrutiny and refinement of therapeutic strategies to address the dynamic needs of patients with HR+/HER2-low mBC.

## Conclusions

Our real-world study brings into focus pivotal facets of the treatment landscape for individuals diagnosed with HR+/HER2-low mBC receiving CT. Though most patients received CT after completing at least 1 HT-based regimen, about one-fourth of patients managed in the community practice setting initiate CT as 1L therapy likely due to high disease burden and worse clinical pathological characteristics. The clinical outcomes associated with CT were suboptimal with median PFS of less than 10 months. Consequently, our study highlights a critical gap in available therapeutic alternatives to CT in early lines of therapy, underscoring the pressing need for innovative and more efficacious treatment options to meet the evolving needs of patients navigating HR+/HER2-low mBC. The advent of HER2-targeted treatment for this indication will likely help to address this gap, with T-DXd potentially representing an alternative for patients in need of more effective treatment in early lines of therapy.

## Supplementary Material

oyaf106_suppl_Supplementary_Tables_1-7_Figures_1-3

## Data Availability

The data that support the findings of this study are not openly available due to reasons of sensitivity and privacy of the data sources.
