# Supplementary material for: Real-world treatment patterns and outcomes of patients with hormone receptor-positive/HER2-low metastatic breast cancer treated with chemotherapy
Source: Oncologist. 2025 Jun 17;30(6):oyaf106. doi: 10.1093/oncolo/oyaf106 (PMC12200229; doi:10.1093/oncolo/oyaf106)
Supplement: oyaf106_suppl_Supplementary_Tables_1-7_Figures_1-3 [file oyaf106_suppl_supplementary_tables_1-7_figures_1-3.docx]

**Real-World Treatment Patterns and Outcomes of Patients With HER2-low/Hormone Receptor-Positive Metastatic Breast Cancer Treated With Chemotherapy**

**SUPPLEMENT**

Contents

[**Supplementary Figure 1: Flow Chart** 2](#_Toc170385762)

[**Supplementary Figure 2. Kaplan Meier Curves of rwPFS in Patients With HR+/HER2-low Disease** 3](#_Toc170385763)

[**2a: KM Curves for Patients With HR+/HER2-low Disease, N=223 pts** 3](#_Toc170385764)

[**2b: KM Curves for Patients Who Received 1L CT and CT After an HT-Based Regimen, (n= 45 Versus n=178)** 4](#_Toc170385765)

[**Supplementary Figure 3: Kaplan Meier Curves for Time to Treatment Discontinuation** 5](#_Toc170385766)

[**3a: KM Curves for Patients With HR+ HER2-low Disease, N=223** 5](#_Toc170385767)

[**3b: KM Curves for Patients Who Received 1L CT and CT After an HT-Based Regimen (n=45 Versus n=178)** 6](#_Toc170385768)

[**Supplementary Table 1: Key Data Elements Collected** 7](#_Toc170385769)

[**Supplementary Table 2: Data Quality Assurance and Quality Control** 8](#_Toc170385770)

[**Supplementary Table 3: Patient Selection** 9](#_Toc170385771)

[**Supplementary Table 4: Physician and Practice Characteristics, N=33** 10](#_Toc170385772)

[**Supplementary Table 5: Demographic and Clinical Characteristics of All Patients Included in Data Collection, N=444** 12](#_Toc170385773)

[**Supplementary Table 6: Treatment Patterns Based on HR and HER2 Status, n=120 and n=324** 16](#_Toc170385774)

[**Supplementary Table 7. Patient Characteristics Among Patients with HR-positive/HER2-low1 Disease, N=223** 17](#_Toc170385775)

# **Supplementary Figure 1: Flow Chart**


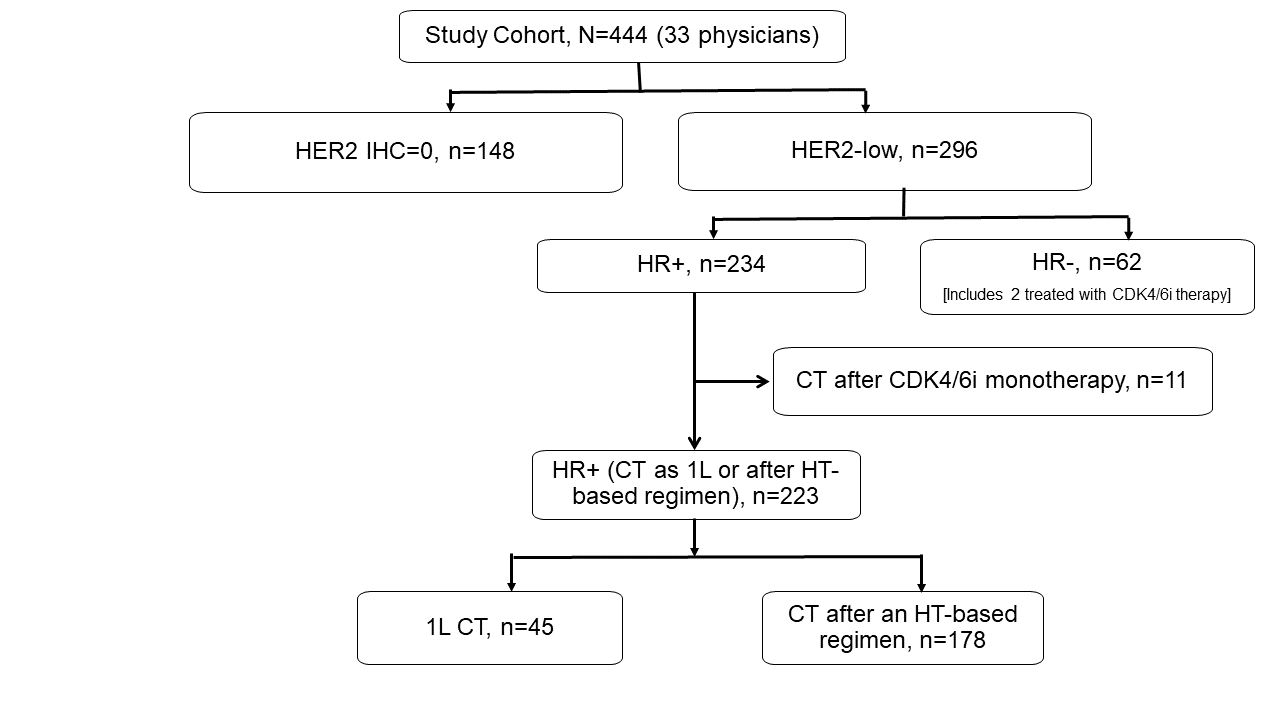


**Abbreviations:** 1L= first-line; CDK4/6= cyclin dependent kinase 4/6; CDK4/6i= CDK4/6 inhibitor; CI= confidence interval; CT= chemotherapy; HER2= human epidermal growth factor receptor 2; HR= hormone receptor; HT= hormonal therapy; IHC= immunohistochemical.

# **Supplementary Figure 2. Kaplan Meier Curves of rwPFS in Patients With HR+/HER2-low Disease**

## **2a: KM Curves for Patients With HR+/HER2-low Disease, N=223 pts**


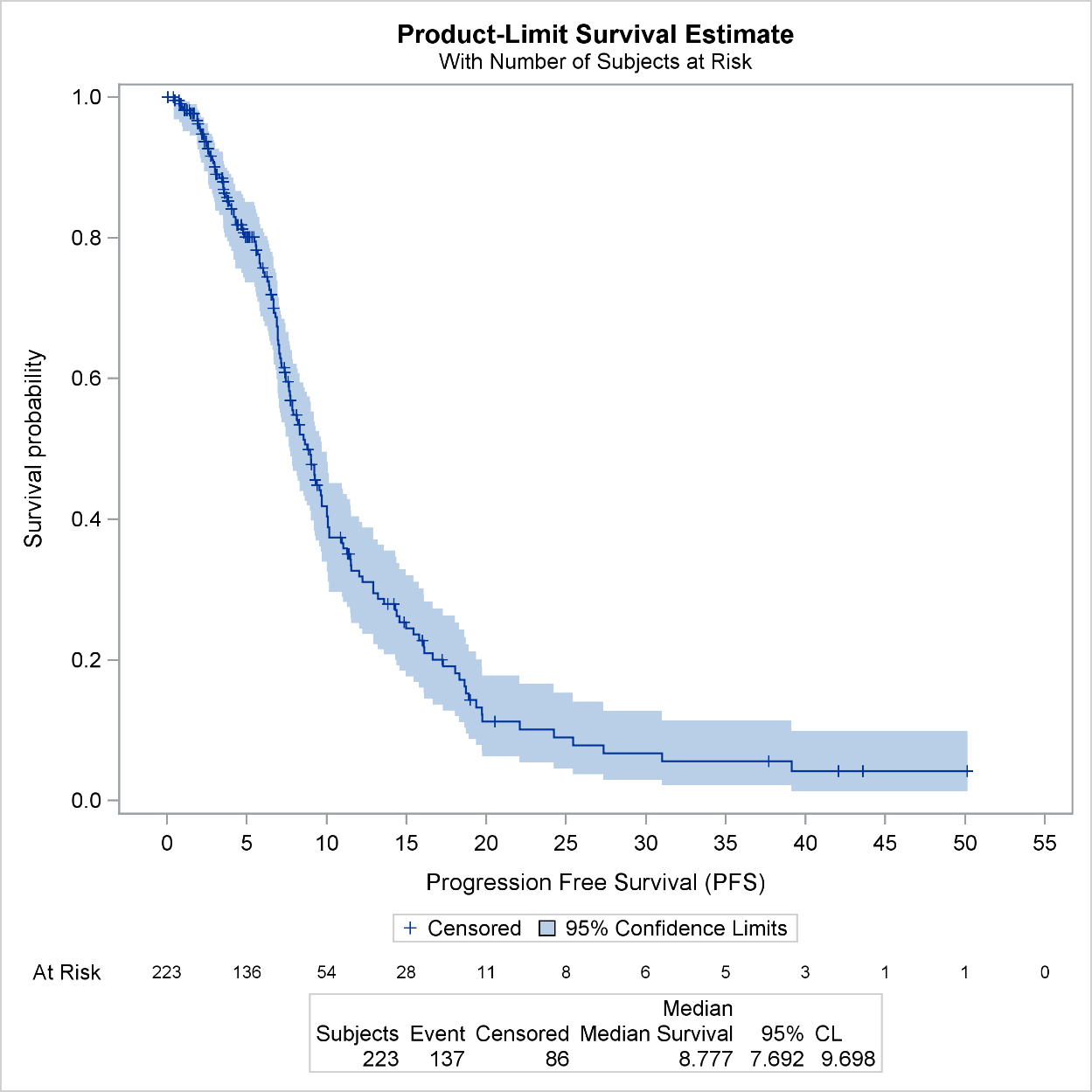


**Abbreviations:** CL= confidence limits; HER2= human epidermal growth factor receptor 2; HR= hormone receptor; KM= Kaplan-Meier.

## **2b: KM Curves for Patients Who Received 1L CT and CT After an HT-Based Regimen, (n= 45 Versus n=178)**


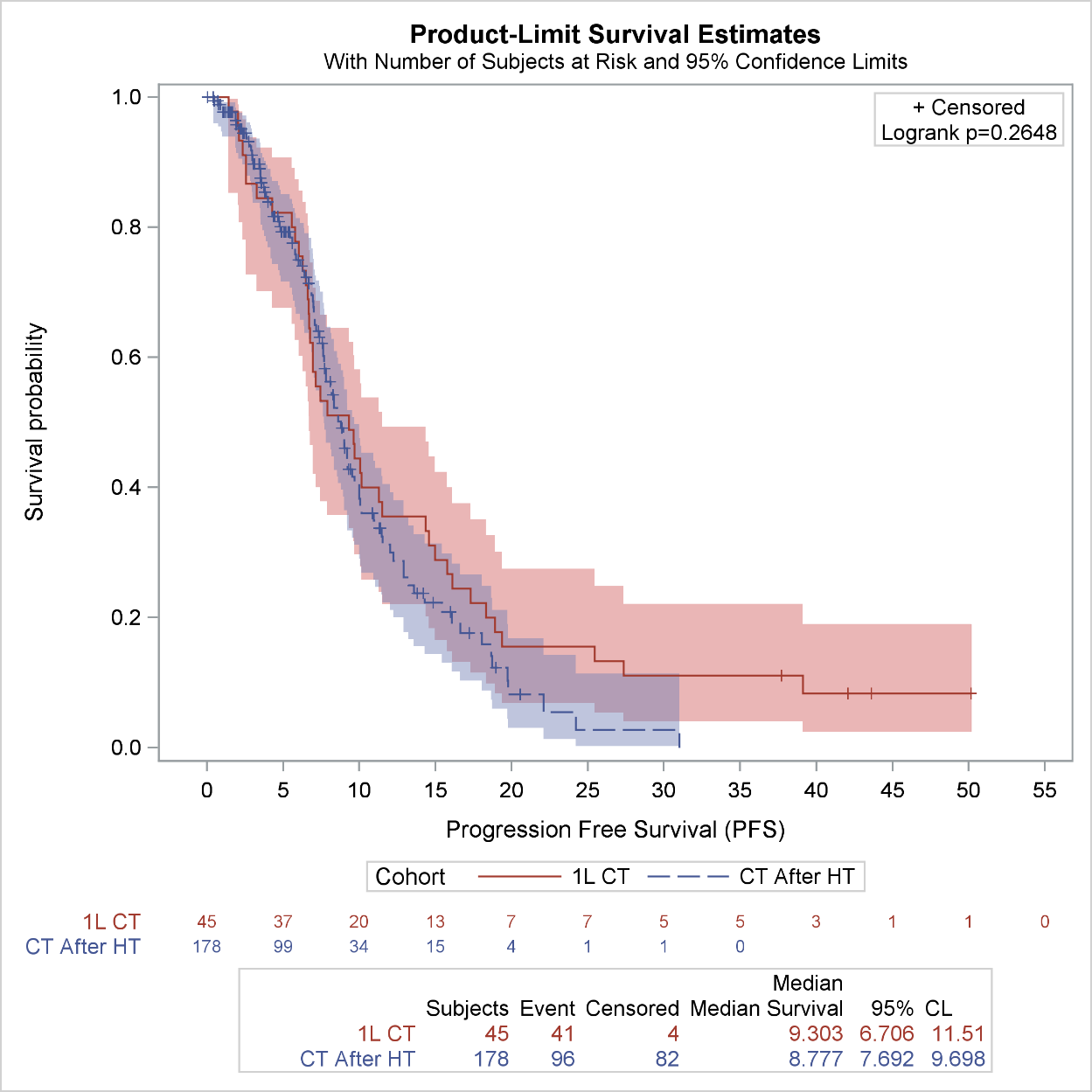


**Notes:** Kaplan-Meier plots of time from initiation of therapy to first reported disease progression or death. Patients who discontinued treatment for a reason other than disease progression or death were censored at discontinuation date and patients still on treatment at the time of data collection were censored at date of last encounter.

**Abbreviations:** 1L= first‑line; CL= confidence limits; CT= chemotherapy; HER2= human epidermal growth factor receptor 2; HT= hormonal therapy; KM= Kaplan-Meier.

# **Supplementary Figure 3: Kaplan Meier Curves for Time to Treatment Discontinuation**

## **3a: KM Curves for Patients With HR+ HER2-low Disease, N=223**


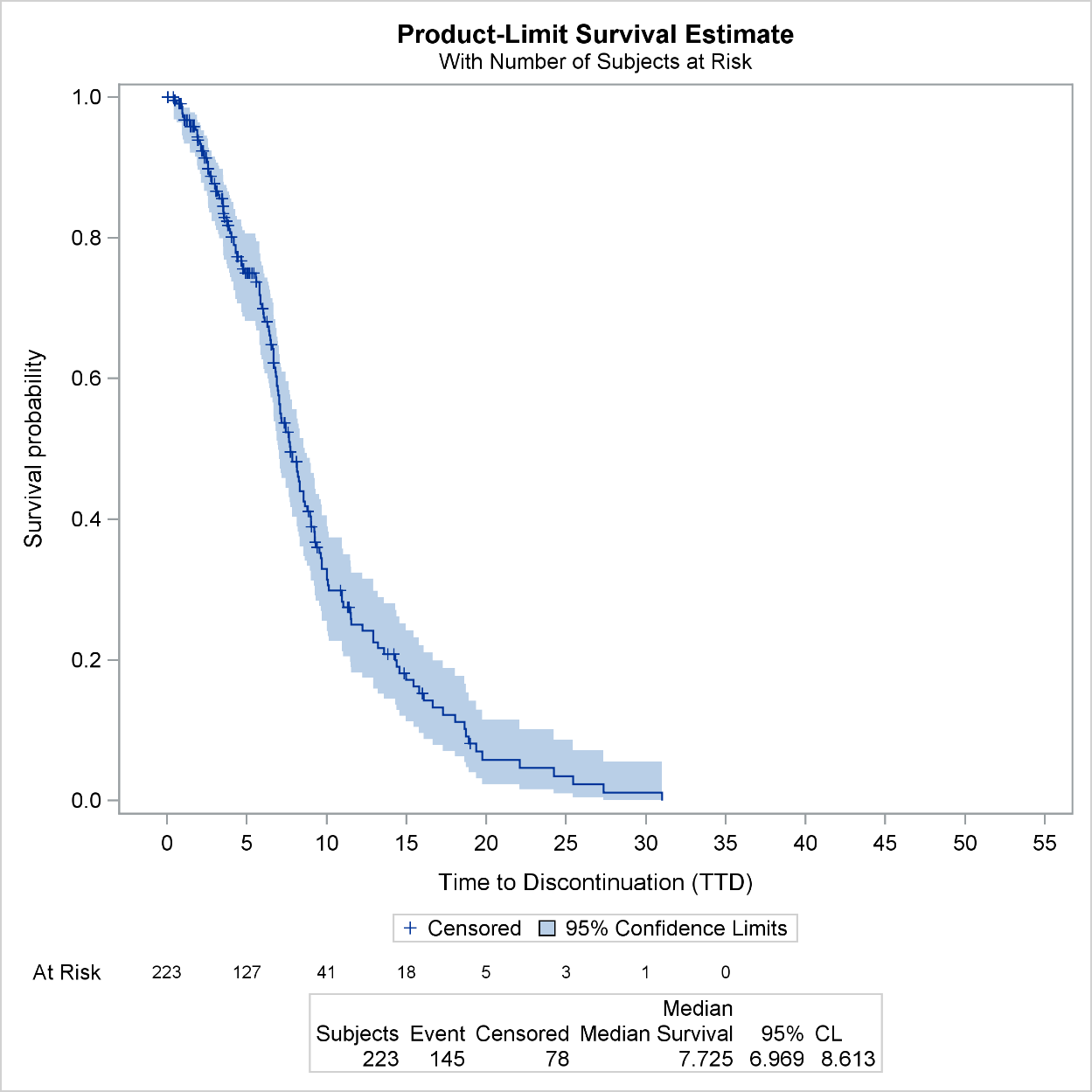


**Abbreviations:** CL= confidence limits; HER2= human epidermal growth factor receptor 2; HT= hormonal therapy; KM= Kaplan-Meier.

## **3b: KM Curves for Patients Who Received 1L CT and CT After an HT-Based Regimen (n=45 Versus n=178)**


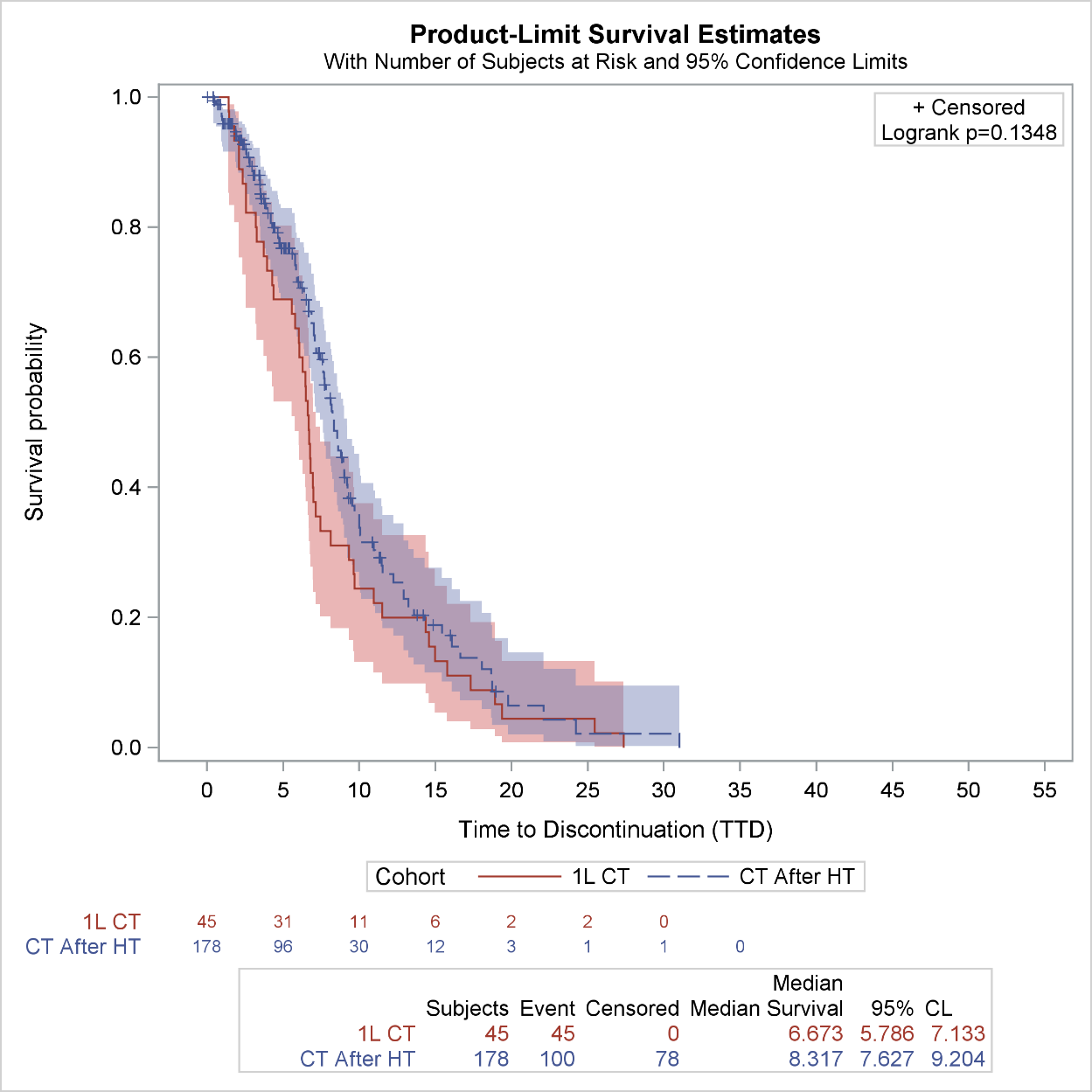


**Notes:** Kaplan-Meier plots of time from initiation to discontinuation of line of therapy or date of death, whichever occurred first. Patients who did not discontinue therapy were censored at last encounter.

**Abbreviations:** 1L= first‑line; CL= confidence limits; CT= chemotherapy; HT= hormonal therapy; KM= Kaplan-Meier.

# **Supplementary Table 1: Key Data Elements Collected**

| **Provider Characteristics** | **Demographic/Clinical Characteristics** | **Treatment Regimens** | **Response, Progression, Survival** |
| --- | --- | --- | --- |
| - Primary practice setting (e.g., private community vs. academic, geographic region, urban vs. rural vs. suburban) | - Sex, year of birth, insurance, geographic region of residence, race/ethnicity | - Systemic treatments received and initiation/discontinuation dates | - Date of first response/date of best response |
| - Years in practice | - Date of initial breast cancer diagnosis | - Rationale for treatment discontinuation | - Disease response |
| - Specialty | - Medical history |  | - Progression date based on provider report |
| - Estimated number of mBC patients seen/treated per typical month | - Date of mBC diagnosis |  | - Date of last follow-up |
|  | - Sites of metastases, lymph node involvement |  | - Date of death/cause of death |
| - HER2 testing patterns and accessibility of results | - Hormone receptor and HER2 status |  |  |
|  | - Eastern Cooperative Oncology Group Performance Status |  |  |
| **Abbreviations:** HER2= human epidermal growth factor receptor 2; mBC= metastatic breast cancer. | | | |

# **Supplementary Table 2: Data Quality Assurance and Quality Control**

| All submitted electronic case report forms (eCRFs) were subject to multiple data quality assurance (QA) and quality control (QC) measures. During data collection, the Cardinal Health Research Operations team reviewed all submitted eCRFs, inspecting implausible dates (e.g., date of death prior to last date of treatment), non-standard treatments (e.g., treatment regimens unknown to be used for the disease under study), lab and radiology results inconsistent with known clinical parameters, and other clinical data inconsistent with known standards and outcomes. |
| --- |
| Data queries generated during this process were maintained in the study QC log. In addition to review of the submitted data, the study statistician conducted an analysis to identify any data points inconsistent with the study population average (outliers). This included a descriptive analysis of the provider characteristics, demographics, baseline clinical and disease characteristics, and characteristics of treatment patterns (e.g., duration of therapy). The eCRFs containing outlier data were flagged and recorded in the QC log and delivered to the Research Operations team. |
| The Cardinal Health Research Operations team generated queries for physicians based on data from the QC log. Physicians were subsequently contacted for query resolution and/or data validation. Participating physicians were asked to create a 4-digit unique identifier code per patient, which was transmitted to Cardinal Health through the eCRF and used for identifying the patient record for validation between Cardinal Health and the provider. Individual eCRFs that could not be validated were removed from the study dataset. All other data were considered valid, provided additional patients from that physician were not selected for random validation. |
| Queries were also generated randomly. Random QC occurred by selecting an eCRF from each provider. The physician was asked to complete a 3-data point validation exercise for the patient, whereby the physician was given the unique patient identifier but no other information. The physician was asked to re-enter the data elements. A physician who failed to validate all data points for a selected patient was required to submit to further clinical data review. Patients for whom the data were deemed questionable by Cardinal Health were excluded. No resampling to replace the excluded eCRF was performed. Any physician who was non-responsive to a validation request had all submitted data excluded from the study. |

# **Supplementary Table 3: Patient Selection**

| Inclusion Criteria   - - Age 18+ at diagnosis of mBC   - Received chemotherapy for the treatment of mBC   - Received at least two lines of systemic therapy for mBC with the first line initiated between February 19, 2016, and December 31, 2018   - Must fit in one of the following categories based on their most recent HER2 testing results:     - HR negative: IHC 0; IHC 1+; IHC 2+ and FISH/ISH negative     - HR positive:       - IHC 0       - Received prior CDK4/6-targeted treatment for mBC - IHC 1+; IHC 2+ and FISH/ISH negative       - Received no prior CDK4/6-targeted treatment for mBC - IHC 1+; IHC 2+ and FISH/ISH negative |
| --- |
| Exclusion Criteria   - - Participation in any clinical trial for mBC |
| **Abbreviations:** CDK4/6= cyclin dependent kinase 4/6; FISH= fluorescence in situ hybridization; HER2= human epidermal growth factor receptor 2; HR= hormone receptor; IHC= immunohistochemistry; ISH= in situ hybridization; mBC= metastatic breast cancer. |

# **Supplementary Table 4: Physician and Practice Characteristics, N=33**

|  | **N=33** |
| --- | --- |
| **Primary practice setting (n, %)** |  |
| Solo practitioner | 0 (0) |
| Small private community practice (2-5 physicians) | 8 (24.2) |
| Medium-sized private community practice (6-10 physicians) | 8 (24.2) |
| Large private community practice (>10 physicians) | 11 (33.3) |
| Community practice owned by an academic center | 1 (3.0) |
| Academic medical center | 2 (6.1) |
| Affiliated teaching hospital | 3 (9.1) |
| VA/military hospital/DOD | 0 (0) |
| Other | 0 (0) |
| **Primary US practice region (n, %)^1^** |  |
| Northeast | 8 (24.2) |
| Midwest | 5 (15.2) |
| South | 11 (33.3) |
| West | 9 (27.3) |
| **Urbanicity of primary practice (n, %)** |  |
| Urban | 16 (48.5) |
| Suburban | 12 (36.4) |
| Rural | 5 (15.2) |
| **Years in practice** |  |
| Mean, SD | 16.7 (6.2) |
| Min, max | 5.0 (28.0) |
| Median, p25-p75 | 15.0 (12.0-20.0) |
| **Provider board-certified/-eligible medical specialty (n, %)^2^** |  |
| Medical oncology | 17 (51.5) |
| Hematology | 3 (9.1) |
| Hematology/Oncology | 19 (57.6) |
| Other | 0 (0) |
| **Estimated number of mBC patients seen/treated per typical month^3^** |  |
| **Total (range 0-250)** |  |
| Mean, SD | 44.7 (31.5) |
| Min, max | 12.0 (175.0) |
| Median, p25-p75 | 35.0 (28.0-48.0) |
| **HER2-positive** |  |
| Mean, SD | 15.1 (13.0) |
| Min, max | 4.0 (65.0) |
| Median, p25-p75 | 10.0 (8.0-15.0) |
| **HER2-negative** |  |
| Mean, SD | 29.6 (24.9) |
| Min, max | 5.0 (150.0) |
| Median, p25-p75 | 25.0 (20.0-32.0) |
| **Patient HER2 testing and accessibility of results** |  |
| **Typical setting of HER2 testing (n, %)^2^** |  |
| **IHC** |  |
| In-house lab | 22 (66.7) |
| External reference lab | 16 (48.5) |
| Other | 0 (0) |
| **FISH/ISH** |  |
| In-house lab | 12 (36.4) |
| External reference lab | 24 (72.7) |
| Other | 0 (0) |
| **Typical timing of HER2 FISH/ISH testing relative to HER2 IHC testing (n, %)^2^** |  |
| At the same time that HER2 IHC testing is ordered | 10 (30.3) |
| Following HER2 IHC results of 1+ | 5 (15.2) |
| Following HER2 IHC results of 2+ | 25 (75.8) |
| Other: | 1 (3.0) |
| Before also, sometimes | 1 (3.0) |
| **Typical visibility and location of HER2 test results in EMR (n, %)** |  |
| HR status |  |
| Scanned pathology or lab report only | 9 (27.3) |
| Manually entered into EMR patient record | 7 (21.2) |
| Automatically populated from pathology report into EMR | 17 (51.5) |
| Other | 0 (0) |
| HER2 status |  |
| Scanned pathology or lab report only | 9 (27.3) |
| Manually entered into EMR patient record | 7 (21.2) |
| Automatically populated from pathology report into EMR | 17 (51.5) |
| Other | 0 (0) |
| IHC score |  |
| Scanned pathology or lab report only | 10 (30.3) |
| Manually entered into EMR patient record | 7 (21.2) |
| Automatically populated from pathology report into EMR | 16 (48.5) |
| Other | 0 (0) |
| FISH/ISH result |  |
| Scanned pathology or lab report only | 11 (33.3) |
| Manually entered into EMR patient record | 7 (21.2) |
| Automatically populated from pathology report into EMR | 15 (45.5) |
| Other | 0 (0) |
| **Notes:** ^1^**Northeast** includes Connecticut, Delaware, Massachusetts, Maine, Maryland, New Hampshire, New Jersey, New York, Pennsylvania, Rhode Island, Vermont; **Midwest** includes Iowa, Illinois, Indiana, Kansas, Michigan, Minnesota, Missouri, North Dakota, Nebraska, Ohio, South Dakota, Wisconsin; **South** includes Alabama, Arkansas, District of Columbia, Florida, Georgia, Kentucky, Louisiana, Mississippi, North Carolina, Oklahoma, South Carolina, Tennessee, Texas, Virginia, West Virginia; **West** includes Alaska, Arizona, California, Colorado, Hawaii, Idaho, Montana, New Mexico, Nevada, Oregon, Utah, Washington, Wyoming.  ^2^Not mutually exclusive.  ^3^Irrespective of study eligibility (range 0-250). | |
| **Abbreviations:** DOD= Department of Defense; EMR= electronic medical record; FISH= fluorescent in situ hybridization; HER2= human epidermal growth factor receptor 2; HR= hormone receptor IHC=immunohistochemistry; ISH= in situ hybridization; max= maximum; mBC= metastatic breast cancer; min= minimum; p25-p75= interquartile range; SD= standard deviation (25^th^ and 75^th^ denote the 25^th^ and 75^th^ percentiles, respectively, in the interquartile range); US= United States; VA= Veteran’s Affairs. | |

# **Supplementary Table 5: Demographic and Clinical Characteristics of All Patients Included in Data Collection, N=444**

|  | **All**  **N=444** | **HER2, IHC=0**  **n= 148** | **HER2-low^1^**  **n= 296** | **p** |  |  |
| --- | --- | --- | --- | --- | --- | --- |
| **Sex at birth (n, %)** |  |  |  | 1.0^a^ |  |  |
| Female | 443 (99.8) | 148 (100) | 295 (99.7) |  |  |  |
| Male | 1 (0.2) | 0 (0) | 1 (0.3) |  |  |  |
| **Race (n, %)** |  |  |  | 0.60^a^ |  |  |
| White | 282 (63.5) | 98 (66.2) | 184 (62.2) |  |  |  |
| Black/African American | 114 (25.7) | 38 (25.7) | 76 (25.7) |  |  |  |
| Asian | 28 (6.3) | 8 (5.4) | 20 (6.8) |  |  |  |
| Native Hawaiian or Other Pacific Islander | 5 (1.1) | 2 (1.4) | 3 (1.0) |  |  |  |
| American Indian or Alaska Native | 4 (0.9) | 1 (0.7) | 3 (1.0) |  |  |  |
| Unknown^2^ | 11 (2.5) | 1 (0.7) | 10 (3.4) |  |  |  |
| **Ethnicity (n, %)** |  |  |  | 0.22^a^ |  |  |
| Hispanic/Latino/Latina | 63 (14.2) | 22 (14.9) | 41 (13.9) |  |  |  |
| Non-Hispanic/Non-Latino/Non-Latina | 377 (84.9) | 125 (84.5) | 252 (85.1) |  |  |  |
| Unknown | 4 (0.9) | 1 (0.7) | 3 (1.0) |  |  |  |
| **Patient US residence region (n, %)^3^** |  |  |  | 0.91^a^ |  |  |
| Northeast | 111 (25.0) | 33 (22.3) | 78 (26.4) |  |  |  |
| Midwest | 69 (15.5) | 20 (13.5) | 49 (16.6) |  |  |  |
| South | 150 (33.8) | 58 (39.2) | 92 (31.1) |  |  |  |
| West | 114 (25.7) | 37 (25.0) | 77 (26.0) |  |  |  |
| **Most recent primary insurance (n, %)** |  |  |  | 0.36^b^ |  |  |
| Commercial | 199 (44.8) | 70 (47.3) | 129 (43.6) |  |  |  |
| Medicare | 197 (44.4) | 59 (39.9) | 138 (46.6) |  |  |  |
| Medicaid | 44 (9.9) | 19 (12.8) | 25 (8.4) |  |  |  |
| Military | 1 (0.2) | 0 (0.0) | 1 (0.3) |  |  |  |
| Self-pay | 2 (0.5) | 0 (0.0) | 2 (0.7) |  |  |  |
| Unknown | 0 (0.0) | 0 (0.0) | 0 (0.0) |  |  |  |
| Other: | 1 (0.2) | 0 (0.0) | 1 (0.3) |  |  |  |
| *Medicare Advantage* | 1 (0.2) | 0 (0.0) | 1 (0.3) |  |  |  |
| **Age at initial BC diagnosis, years** |  |  |  | 0.85^c^ |  |  |
| Mean, SD | 60 (10.8) | 60 (11.13) | 60 (10.7) |  |  |  |
| Min, max | 27 (89.0) | 36 (89.00) | 27 (88.0) |  |  |  |
| Median, p25-p75 | 61 (52-68) | 61 (52-68) | 61 (52-68) |  |  |  |
| **Age at mBC diagnosis, years** |  |  |  | 0.51^c^ |  |  |
| Mean, SD | 60 (10.9) | 61 (11.7) | 60 (10.8) |  |  |  |
| Min, max | 34 (85.0) | 38 (85.0) | 34 (80.0) |  |  |  |
| Median, p25-p75 | 60 (52-68) | 62 (52.5-68.5) | 60 (52-67) |  |  |  |
| **Smoking status (n, %)** |  |  |  | 0.49^b^ |  |  |
| Never smoked | 245 (55.2) | 87 (58.8) | 158 (53.4) |  |  |  |
| Current smoker | 37 (8.3) | 10 (6.8) | 27 (9.1) |  |  |  |
| History of smoking | 162 (36.5) | 51 (34.5) | 111 (37.5) |  |  |  |
| **Anatomic site of metastasis at diagnosis of mBC (n, %)^4,5^** |  |  |  |  |  |  |
| Visceral^6^ | 46 (10.4) | 16 (10.8) | 30 (10.1) |  |  |  |
| Adrenal gland | 30 (6.8) | 9 (6.1) | 21 (7.1) | 0.82^b^ |  |  |
| Brain | 63 (14.2) | 23 (15.5) | 40 (13.5) | 0.69^b^ |  |  |
| Local lymph node(s) | 109 (24.5) | 38 (25.7) | 71 (24.0) | 0.56^b^ |  |  |
| Regional/Distal lymph node(s) | 28 (6.3) | 7 (4.7) | 21 (7.1) | 0.70^b^ |  |  |
| Skin/soft tissue | 10 (2.3) | 3 (2.0) | 7 (2.4) | 0.33^b^ |  |  |
| Gastrointestinal system | 1 (0.2) | 0 (0.0) | 1 (0.3) | 1.00^a^ |  |  |
| Genitourinary system | 9 (2.0) | 3 (2.0) | 6 (2.0) | 1.00^a^ |  |  |
| Ovary | 0 (0.0) | 0 (0.0) | 0 (0.0) | 1.00^a^ |  |  |
| Gynecologic system (excluding ovary) | 164 (36.9) | 55 (37.2) | 109 (36.8) | NA |  |  |
| Liver | 232 (52.3) | 73 (49.3) | 159 (53.7) | 0.94^b^ |  |  |
| Lung | 45 (10.1) | 9 (6.1) | 36 (12.2) | 0.38^b^ |  |  |
| Pleura, pericardial, and/or peritoneal cavity | 1 (0.2) | 0 (0.0) | 1 (0.3) | 0.045^b^ |  |  |
| Other: Bone marrow | 1 (0.2) | 0 (0.0) | 1 (0.3) | 1.00^a^ |  |  |
| Bony |  |  |  |  |  |  |
| Bone | 299 (67.3) | 96 (64.9) | 203 (68.6) | 0.43^b^ |  |  |
| **Number of distinct anatomic sites of metastasis (n, %)** |  |  |  | 0.33^b^ |  |  |
| 0 to 1 | 130 (29.3) | 50 (33.8) | 80 (27.0) |  |  |  |
| 2 to 3 | 245 (55.2) | 76 (51.4) | 169 (57.1) |  |  |  |
| 4+ | 69 (15.5) | 22 (14.9) | 47 (15.9) |  |  |  |
| **Comorbidities/chronic conditions at mBC diagnosis (n, %)^4,5^** |  |  |  |  |  |  |
| AIDS/HIV | 2 (0.5) | 1 (0.7) | 1 (0.3) | 1.00^a^ |  |  |
| Cardiovascular disease | 67 (15.1) | 23 (15.5) | 44 (14.9) | 0.85^b^ |  |  |
| Cerebrovascular disease | 22 (5.0) | 9 (6.1) | 13 (4.4) | 0.44^b^ |  |  |
| Chronic pulmonary disease | 60 (13.5) | 21 (14.2) | 39 (13.2) | 0.77^b^ |  |  |
| Congestive heart failure | 16 (3.6) | 2 (1.4) | 14 (4.7) | 0.072^b^ |  |  |
| Connective tissue disease | 21 (4.7) | 8 (5.4) | 13 (4.4) | 0.64^b^ |  |  |
| Dementia | 6 (1.4) | 2 (1.4) | 4 (1.4) | 1.00^a^ |  |  |
| Depression | 90 (20.3) | 31 (20.9) | 59 (19.9) | 0.80^b^ |  |  |
| Diabetes with chronic complications | 42 (9.5) | 13 (8.8) | 29 (9.8) | 0.73^b^ |  |  |
| Diabetes without chronic complications | 74 (16.7) | 24 (16.2) | 50 (16.9) | 0.86^b^ |  |  |
| Hemiplegia or paraplegia | 1 (0.2) | 1 (0.7) | 0 (0.0) | 0.33^a^ |  |  |
| Hypertension | 228 (51.4) | 70 (47.3) | 158 (53.4) | 0.23^b^ |  |  |
| Liver disease - mild | 16 (3.6) | 3 (2.0) | 13 (4.4) | 0.21^b^ |  |  |
| Liver disease - moderate or severe | 0 (0.0) | 0 (0.0) | 0 (0.0) | NA |  |  |
| Myocardial infarction | 19 (4.3) | 5 (3.4) | 14 (4.7) | 0.51^b^ |  |  |
| Other hematologic malignancy | 2 (0.5) | 0 (0.0) | 2 (0.7) | 0.55^a^ |  |  |
| Other non-hematologic malignancy | 2 (0.5) | 0 (0.0) | 2 (0.7) | 0.55^a^ |  |  |
| Peptic ulcer disease | 38 (8.6) | 11 (7.4) | 27 (9.1) | 0.55^b^ |  |  |
| Peripheral vascular disease | 10 (2.3) | 1 (0.7) | 9 (3.0) | 0.18^a^ |  |  |
| Renal disease | 47 (10.6) | 11 (7.4) | 36 (12.2) | 0.13^b^ |  |  |
| Rheumatologic disease | 29 (6.5) | 6 (4.1) | 23 (7.8) | 0.14^b^ |  |  |
| Thromboembolic events (arterial or venous) | 21 (4.7) | 5 (3.4) | 16 (5.4) | 0.34^b^ |  |  |
| None of the above | 94 (21.2) | 36 (24.3) | 58 (19.6) | 0.25^b^ |  |  |
| Other: | 12 (2.7) | 3 (2.0) | 9 (3.0) | 0.76^a^ |  |  |
| *Asthma* | 2 (0.5) | 0 (0.0) | 2 (0.7) |  |  |  |
| *HTN* | 1 (0.2) | 0 (0.0) | 1 (0.3) |  |  |  |
| *Hypothyroidism* | 1 (0.2) | 0 (0.0) | 1 (0.3) |  |  |  |
| *hypothyroidism, migraine headaches* | 1 (0.2) | 0 (0.0) | 1 (0.3) |  |  |  |
| *hyperlipidemia, major depression* | 1 (0.2) | 0 (0.0) | 1 (0.3) |  |  |  |
| **Duration of follow-up since initiation of first line (months)^7^** |  |  |  |  |  |  |
| mean, SD | 36.7 (14.1) | 35.0 (15.2) | 37.6 (13.5) | 0.059^d^ |  |  |
| min, max | 4.4 (68.8) | 7.7 (68.8) | 4.4 (68.5) |  |  |  |
| median, p25-p75 | 37.4 (28.9-44.7) | 36.1 (22.0-45.0) | 37.6 (31.0-44.7) |  |  |  |
| **Duration of follow-up since initiation of second line (months)^7^** |  |  |  |  |  |  |
| mean, SD | 19.0 (10.1) | 17.0 (10.3) | 20.0 (9.9) | 0.003^d^ |  |  |
| min, max | 1.5 (55.1) | 2.2 (43.7) | 1.5 (55.1) |  |  |  |
| median, p25-p75 | 17.8 (11.1-25.5) | 15.4 (8.7-23.9) | 18.6 (12.8-26.2) |  |  |  |
| **Duration of follow-up since initiation of third line (months)^7^** |  |  |  |  |  |  |
| mean, SD | 8.3 (7.3) | 7.9 (6.8) | 8.5 (7.4) | 0.44^d^ |  |  |
| min, max | 0.0 (38.5) | 0.4 (36.6) | 0.0 (38.5) |  |  |  |
| median, p25-p75 | 5.9 (3.1-11.6) | 5.6 (3.4-10.3) | 6.3 (3.1-11.7) |  |  |  |
| **Notes:** ^1^HER2-low defined as IHC 1+ or IHC 2+ and FISH/ISH negative.  ^2^Race was entered as Hispanic via open text. However, Hispanic is considered as ethnicity rather than race, and therefore race is unknown.  ^3^**Northeast** includes Connecticut, Delaware, Massachusetts, Maine, Maryland, New Hampshire, New Jersey, New York, Pennsylvania, Rhode Island, Vermont; **Midwest** includes Iowa, Illinois, Indiana, Kansas, Michigan, Minnesota, Missouri, North Dakota, Nebraska, Ohio, South Dakota, Wisconsin; **South** includes Alabama, Arkansas, District of Columbia, Florida, Georgia, Kentucky, Louisiana, Mississippi, North Carolina, Oklahoma, South Carolina, Tennessee, Texas, Virginia, West Virginia; **West** includes Alaska, Arizona, California, Colorado, Hawaii, Idaho, Montana, New Mexico, Nevada, Oregon, Utah, Washington, Wyoming.  ^4^Not mutually exclusive.  ^5^Additional response options that were presented to physicians but were not selected for any patients are excluded here but can be viewed in the final results compendium.  ^6^Defined as metastases in the adrenal gland; GI system; GU system; ovary, gynecological system; liver; lung; pleura, pericardial, and/or peritoneal cavity.  ^a^Fisher’s test.  ^b^Chi-square.  ^c^T test-pooled.  ^d^Monte Carlo Estimate.  **Abbreviations:** AIDS= acquired immunodeficiency syndrome; BC= breast cancer; FISH= fluorescence in situ hybridization; GI= gastrointestinal tract; GU= genitourinary; HER2= human epidermal growth factor receptor 2; HIV= human immunodeficiency virus; HTN= hypertension; IHC= immunohistochemistry; ISH= in situ hybridization; max= maximum; mBC= metastatic breast cancer; min= minimum; NA= not applicable; p25-p75= interquartile range (25th and 75th denote the 25th and 75th percentiles, respectively, in the interquartile range); SD= standard deviation; US= United States. | | | | |  |  |

# **Supplementary Table 6: Treatment Patterns Based on HR and HER2 Status, n=120 and n=324**

|  | **HR-** | | | | **HR+** | | | |
| --- | --- | --- | --- | --- | --- | --- | --- | --- |
|  | **All, n=120** | **HER2, IHC=0**  **n= 58** | **HER2-low^1^**  **n= 62** | **p** | **All,**  **n= 324** | **HER2, IHC=0**  **n= 90** | **HER2-low^1^**  **n= 234** | **p** |
| **Regimen types received for 1L treatment of mBC, n (%)** | | | | | | | | |
| CDK4/6 inhibitor | (0.80) | 0 (0) | 1 (1.60) | 1.00^a^ | 25 (7.70) | 5 (5.60) | 20 (8.50) | 0.19 ^a^ |
| CDK4/6 inhibitor, hormonal | 0 (0) | 0 (0) | 0 (0) |  | 191 (59.0) | 58 (64.4) | 133 (56.8) |  |
| Chemotherapy | 112 (93.0) | 55 (94.8) | 57 (91.9) |  | 50 (15.4) | 9 (10.0) | 41 (17.5) |  |
| Chemotherapy, hormonal | 0 (0) | 0 (0) | 0 (0) |  | 4 (1.20) | 0 (0) | 4 (1.70) |  |
| Chemotherapy, immunotherapy | 4 (3.30) | 2 (3.40) | 2 (3.20) |  | 0 (0) | 0 (0) | 0 (0) |  |
| HER2 targeted | 1 (0.80) | 0 (0) | 1 (1.60) |  | 0 (0) | 0 (0) | 0 (0) |  |
| Hormonal | 2 (1.70) | 1 (1.70) | 1 (1.60) |  | 54 (16.7) | 18 (20.0) | 36 (15.4) |  |
| **Regimen types received for 2L treatment of mBC, n (%)** | | | | | | | | |
| CDK4/6 inhibitor | 0 (0) | 0 (0) | 0 (0) | 0.98^a^ | 5 (1.50) | 3 (3.30) | 2 (0.90) | 0.17^a^ |
| CDK4/6 inhibitor, hormonal | 2 (1.70) | 1 (1.70) | 1 (1.60) |  | 73 (22.5) | 21 (23.3) | 52 (22.2) |  |
| Chemotherapy | 103 (85.8) | 50 (86.2) | 53 (85.5) |  | 93 (28.7) | 24 (26.7) | 69 (29.5) |  |
| Chemotherapy, hormonal | 0 (0) | 0 (0) | 0 (0) |  | 4 (1.20) | 1 (1.10) | 3 (1.30) |  |
| Chemotherapy, immunotherapy | 9 (7.50) | 4 (6.90) | 5 (8.10) |  | 0 (0) | 0 (0) | 0 (0) |  |
| HER2 targeted | 1 (0.80) | 0 (0) | 1 (1.60) |  | 0 (0) | 0 (0) | 0 (0) |  |
| Hormonal | 0 (0) | 0 (0) | 0 (0) |  | 92 (28.4) | 20 (22.2) | 72 (30.8) |  |
| Other targeted | 5 (4.20) | 3 (5.20) | 2 (3.20) |  | 7 (2.20) | 1 (1.10) | 6 (2.60) |  |
| Other targeted, hormonal | 0 (0) | 0 (0) | 0 (0) |  | 50 (15.4) | 20 (22.2) | 30 (12.8) |  |
| **Regimen types received for 3L treatment of mBC, n (% of patients who received 3L)** | | | | | | | | |
| CDK4/6 inhibitor | 0 (0) | 0 (0) | 0 (0) | 0.032^a^ | 1 (0.30) | 1 (1.10) | 0 (0) | 0.035 ^a^ |
| CDK4/6 inhibitor, hormonal | 0 (0) | 0 (0) | 0 (0) |  | 15 (4.60) | 4 (4.40) | 11 (4.70) |  |
| Chemotherapy | 47 (81.0) | 12 (63.2) | 35 (89.7) |  | 244 (75.3) | 78 (86.7) | 166 (70.9) |  |
| Chemotherapy, immunotherapy | 2 (3.40) | 1 (5.30) | 1 (2.60) |  | 1 (0.30) | 0 (0) | 1 (0.40) |  |
| HER2 targeted | 0 (0) | 0 (0) | 0 (0) |  | 21 (6.50) | 2 (2.20) | 19 (8.10) |  |
| Immunotherapy | 0 (0) | 0 (0) | 0 (0) |  | 2 (0.60) | 0 (0) | 2 (0.90) |  |
| Other targeted | 9 (15.5) | 6 (31.6) | 3 (7.70) |  | 13 (4.00) | 1 (1.10) | 12 (5.10) |  |
| Other targeted, hormonal | 0 (0) | 0 (0) | 0 (0) |  | 27 (8.30) | 4 (4.40) | 23 (9.80) |  |
| **Abbreviations:** 1L= first-line; 2L= second-line; 3L= third-line; CDK4/6= cyclin-dependent 4/6; HER2= human epidermal growth factor receptor 2; IHC= immunohistochemistry; HR= hormone receptor; mBC= metastatic breast cancer.  Notes:  ^a^ across all regimen types; Chi-square / Fisher Exact test | | | | | | | | |

# **Supplementary Table 7. Patient Characteristics Among Patients with HR-positive/HER2-low1 Disease, N=223**

|  | **HR+/HER2-Low^1^** | **1L CT** | **CT after an HT-based regimen** |  |
| --- | --- | --- | --- | --- |
|  | **n=223** | **n= 45** | **n= 178** | **p** |
| Smoking status (n, %) |  |  |  | 0.34^c^ |
| Never smoked | 121 (54.3) | 28 (62.2) | 93 (52.2) |  |
| Current smoker | 20 (9.0) | 2 (4.4) | 18 (10.1) |  |
| History of smoking | 82 (36.8) | 15 (33.3) | 67 (37.6) |  |
| Comorbidities/chronic conditions at mBC diagnosis (n, %)^2,3^ |  |  |  |  |
| Cardiovascular disease | 32 (14.3) | 9 (20.0) | 23 (12.9) | 0.23^c^ |
| Cerebrovascular disease | 10 (4.5) | 1 (2.2) | 9 (5.1) | 0.69^b^ |
| Chronic pulmonary disease | 28 (12.6) | 4 (8.9) | 24 (13.5) | 0.41^c^ |
| Congestive heart failure | 14 (6.3) | 3 (6.7) | 11 (6.2) | 1.00^b^ |
| Connective tissue disease | 10 (4.5) | 0 (0.0) | 10 (5.6) | 0.22^b^ |
| Dementia | 2 (0.9) | 0 (0.0) | 2 (1.1) | 1.00^b^ |
| Depression | 40 (17.9) | 9 (20.0) | 31 (17.4) | 0.68^c^ |
| Diabetes with chronic complications | 19 (8.5) | 4 (8.9) | 15 (8.4) | 1.00^b^ |
| Diabetes without chronic complications | 46 (20.6) | 12 (26.7) | 34 (19.1) | 0.26^c^ |
| Hypertension | 127 (57.0) | 29 (64.4) | 98 (55.1) | 0.26^c^ |
| Liver disease – mild | 8 (3.6) | 3 (6.7) | 5 (2.8) | 0.20^b^ |
| Myocardial infarction | 10 (4.5) | 3 (6.7) | 7 (3.9) | 0.43^b^ |
| Other hematologic malignancy | 2 (0.9) | 0 (0.0) | 2 (1.1) | 1.00^b^ |
| Other non-hematologic malignancy | 2 (0.9) | 0 (0.0) | 2 (1.1) | 1.00^b^ |
| Peptic ulcer disease | 20 (9.0) | 4 (8.9) | 16 (9.0) | 1.00^b^ |
| Peripheral vascular disease | 7 (3.1) | 1 (2.2) | 6 (3.4) | 1.00^b^ |
| Renal disease | 26 (11.7) | 3 (6.7) | 23 (12.9) | 0.24^c^ |
| Rheumatologic disease | 18 (8.1) | 2 (4.4) | 16 (9.0) | 0.54^b^ |
| Thromboembolic events (arterial or venous) | 13 (5.8) | 1 (2.2) | 12 (6.7) | 0.47^b^ |
| None of the above | 41 (18.4) | 10 (22.2) | 31 (17.4) | 0.46^c^ |
| Other: | 8 (3.6) | 2 (4.4) | 6 (3.4) | 0.66^b^ |
| *Asthma* | 1 (0.4) | 0 (0.0) | 1 (0.6) |  |
| *HTN* | 1 (0.4) | 0 (0.0) | 1 (0.6) |  |
| *Hypothyroidism* | 4 (1.8) | 2 (4.4) | 2 (1.1) |  |
| *hypothyroidism, migraine headaches* | 1 (0.4) | 0 (0.0) | 1 (0.6) |  |
| *hyperlipidemia, major depression* | 1 (0.4) | 0 (0.0) | 1 (0.6) |  |
| **Notes**: *CT index was defined as1L therapy for the 1L CT group and as the first CT received following an HT-based regimen for the CT after an HT-based regimen group. Patients who received CT post CDK4/6i were excluded.  ^1^HER2-low defined as IHC 1+ or IHC 2+ and FISH/ISH negative.  ^2^Not mutually exclusive.  ^3^Additional response options that were presented to physicians but were not selected for any patients are excluded here.  ^a^T test-Pooled test was used for statistical comparison.  ^b^Fisher’s Exact test was used for statistical comparison.  ^c^Chi-Square test was used for statistical comparison.  **Abbreviations:** 1L= first-line; CDK4/6i= cyclin-dependent 4/6 inhibitor; CT= chemotherapy; FISH= fluorescence in situ hybridization; HER2= human epidermal growth factor receptor 2; HR= hormone receptor; HT= hormonal therapy; HTN= hypertension; IHC= immunohistochemistry; ISH= in situ hybridization; mBC= metastatic breast cancer. | | | | |
